# Versatile Asymmetric Separator with Dendrite‐Free Alloy Anode Enables High‐Performance Li–S Batteries

**DOI:** 10.1002/advs.202202204

**Published:** 2022-06-24

**Authors:** Wenqi Yan, Jin‐Lin Yang, Xiaosong Xiong, Lijun Fu, Yuhui Chen, Zhaogen Wang, Yusong Zhu, Jian‐Wei Zhao, Tao Wang, Yuping Wu

**Affiliations:** ^1^ State Key Laboratory of Materials‐oriented Chemical Engineering Institute of Advanced Materials (IAM) and School of Energy Science and Engineering Nanjing Tech University Nanjing 211816 P. R. China; ^2^ School of Physical and Mathematical Sciences Nanyang Technological University Singapore 637371 Singapore; ^3^ Shenzhen HUASUAN Technology Co. Ltd Shenzhen 518055 P. R. China; ^4^ School of Energy and Environment Southeast University Nanjing 211189 P. R. China

**Keywords:** dendrite‐free anodes, lithium–magnesium alloys, lithium–sulfur batteries, polysulfides shuttling, versatile asymmetric separator

## Abstract

Lithium–sulfur batteries (LSBs) with extremely‐high theoretical energy density (2600 Wh kg^−1^) are deemed to be the most likely energy storage system to be commercialized. However, the polysulfides shuttling and lithium (Li) metal anode failure in LSBs limit its further commercialization. Herein, a versatile asymmetric separator and a Li‐rich lithium–magnesium (Li–Mg) alloy anode are applied in LSBs. The asymmetric separator is consisted of lithiated‐sulfonated porous organic polymer (SPOP‐Li) and Li_6.75_La_3_Zr_1.75_Nb_0.25_O_12_ (LLZNO) layers toward the cathode and anode, respectively. SPOP‐Li serves as a polysulfides barrier and Li‐ion conductor, while the LLZNO functions as an “ion redistributor”. Combining with a stable Li–Mg alloy anode, the symmetric cell achieves 5300 h of Li stripping/plating and the modified LSBs exhibit a long lifespan with an ultralow fading rate of 0.03% per cycle for over 1000 cycles at 5 C. Impressively, even under a high‐sulfur‐loading (6.1 mg cm^−2^), an area capacity of 4.34 mAh cm^−2^ after 100 cycles can still be maintained, demonstrating high potential for practical application.

## Graphical abstract

A versatile asymmetric separator is constructed by covering lithiated sulfonate‐rich porous organic polymer (SPOP‐Li) and Li_6.75_La_3_Zr_1.75_Nb_0.25_O_12_ (LLZNO) on the PP substrate for serving as a LiPSs barrier and an “ion redistributor”, combing with stable Li‐rich Li‐Mg alloy anode, a dendrite‐free anode and high‐energy‐density of LSBs are expected to be realized.

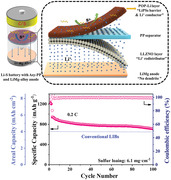

## 1 Introduction

Renewable energy (e.g., wind, tidal, solar, etc.) is critical to achieving carbon neutrality and mitigating the greenhouse effects as an alternative to fossil fuels. Electrochemical energy storage devices are indispensable as an important link in the conversion of renewable energy sources. Especially, lithium–ion batteries (LIBs) have been developed for more than 30 years and are widely used in portable electronics, electric vehicles, etc. due to their high energy density and long lifespan. However, the cutting‐edge commercial LIBs (≈300 Wh kg^−1^) assembled with intercalation electrode materials still have quite a gap to achieve a higher energy density than 500 Wh kg^−1^ based on the Battery500 Consortium plan.^[^ ^1^, ^2^, ^3^ ^]^ Lithium–sulfur batteries (LSBs) with a remarkable high theoretical energy density (2600 Wh kg^−1^) have been proven to offer the opportunity to store nearly 2–4 times as much energy density as LIBs in electrical vehicles today, which are commencing to compete commercially with LIBs.^[^ ^4^, ^5^ ^]^

The operating mechanism of LSBs is a reversible electrochemical conversion process that produces long‐chain and short‐chain lithium polysulfides (LiPSs) between elemental sulfur (S) and Li‐ions (Li^+^).^[^ ^6^, ^7^ ^]^ The practical application of LSBs must handle some notorious problems. For S cathode, the “shuttle effect” is caused by the soluble polysulfides (i.e., molecular LiPSs and polysulfides anions). They often diffuse throughout the separator and then be reduced by the lithium metal (Li) anode, resulting in the irreversible loss of active S cathode and passivation of the Li anode.^[^ ^8^, ^9^ ^]^ Therefore, for tackling the “shuttle effect” of soluble polysulfides, numerous works have contributed to immobilizing the polysulfides via interlayer on separators in LSBs. The interlayer can be composed by carbonaceous materials,^[^ ^10^, ^11^, ^12^ ^]^ metal‐based materials,^[^ ^13^, ^14^, ^15^ ^]^ metal organic frameworks,^[^ ^16^, ^17^ ^]^ covalent organic frameworks,^[^ ^18^, ^19^ ^]^ and so on. However, unlike molecular LiPSs, the polysulfides (PSs) anions are a kind of Lewis base that cannot be entirely trapped by the above materials. Further, the sacrificial Li^+^ conductivity in these layers affects the electrode reaction kinetics and internal resistance.^[^ ^20^, ^21^ ^]^ Therefore, the ideal modified layer should have a superb ability to block the polysulfide species, and simultaneously accelerate Li^+^ migration to form a homogeneous Li^+^ flux to reach the Li anode.

**Figure 1:**
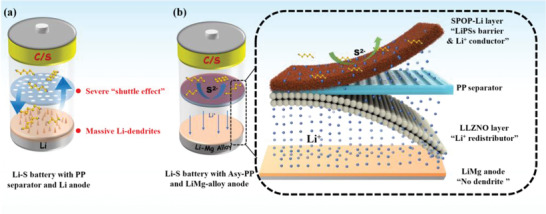
Schematic diagrams of operating LSBs: a) traditional PP separators and Li anode, and b) our prepared asymmetrical separator and Li–Mg alloy anode.

Due to the ultralow redox potential of −3.04 V (*vs*. SHE), Li metal anode inevitably reacts with the liquid electrolyte and forms a solid electrolyte interface (SEI). The fragile self‐formed SEI is continuously broken and repaired during repeated Li stripping/plating. Further, the infinite Li volume change during repeated Li stripping/plating destructs the bulk. The above reasons lead to severe electrolyte/Li consumption, extensive Li pulverization, and massive dendrites growth (**Figure** **1**a). Beyond that, the aggregated PSs and nonuniform Li^+^ flux distribution from the separator could accelerate LSBs deterioration.^[^ ^22^, ^23^ ^]^ Li‐rich lithium–magnesium (Li–Mg) anode with high Li^+^ diffusion coefficients and bulk stabilization after delithiation has been considered as an ideal anode for practical 500 Wh kg^−1^ LSBs.^[^ ^1^, ^24^ ^]^ Therefore, if there is homogeneous Li^+^ deposition on the Li–Mg alloy anode, upon coupling with the excellent interfacial and bulk stability of Li–Mg alloys, there is an opportunity for high‐energy‐density and safe LSBs.

Herein, we endowed LSBs with great potential for practical application by inducing a versatile asymmetric PP separator (Asy‐PP) and a Li‐rich Li–Mg alloy anode (Figure 1b). The asymmetric separator was constructed by covering lithiated sulfonate‐rich porous organic polymer (SPOP‐Li) and Li_6.75_La_3_Zr_1.75_Nb_0.25_O_12_ (LLZNO) on the PP substrate, respectively. The SPOP‐Li with concentrated negative charged sulfonates facing the S cathode could act as an ionic sieve for inhibiting the LiPSs shuttling (electrostatic repulsion of PSs anions and adsorption of molecular LiPSs) and facilitating the Li^+^ migration. Meanwhile, the LLZNO layer toward the anode serves as an “ion redistributor” to regulate Li^+^ flux. Additionally, the Li‐rich Li–Mg alloy anode enhances the stability of magnesium‐containing SEI and the conductive matrix of the Li–Mg alloy allows for the preservation of microstructural and bulk integrity during cycling. Combined with the homogenous Li^+^ distribution, a stable and dendrite‐free anode can be obtained in LSBs. Accordingly, Li–Mg alloy anode with “ion redistributor” can achieve 5300 h of Li stripping/plating in a symmetric cell and a Coulombic efficiency (CE) of above 99% over 320 cycles in a Li–Mg/Cu cell. The S/Asy‐PP/Li–Mg batteries deliver a high initial capacity of 1116 mA g^−1^ and slow decay with an average CE of 99.5% over 400 cycles at 1 C. The long‐term cycling at a high current of 5 C is also achieved with an ultralow fading rate of 0.03% per cycle for over 1000 cycles. Notably, a reversible area capacity of 5.75 mAh cm^−2^ and 4.34 mAh cm^−2^ can be remained over 100 cycles under a high S cathode loading of 6.1 mg cm^−2^.

## 2 Results and Discussion

### 2.1 Structure Characterization and Li^+^ Ion Transport

**Figure 2:**
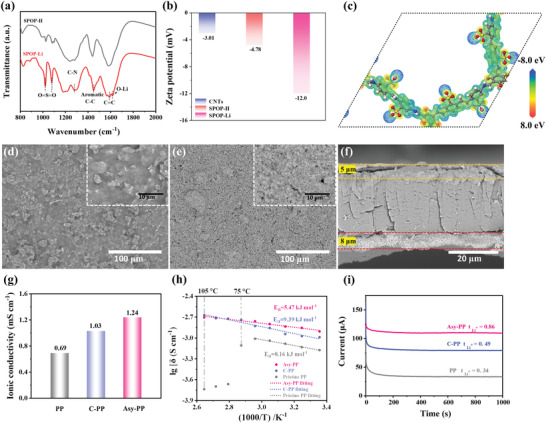
Structure characterization and Li^+^ ion transport. a) FT‐IR of SPOP‐H and SPOP‐Li. b) Zeta potential of the CNTs, SPOP‐H, and SPOP‐Li. c) Electrostatic potential distributions of SPOP‐Li. SEM images: the surface of d) SPOP‐Li layer and e) LLZNO layer, and cross‐section of Asy‐PP. g) The ionic conductivities and h) the Arrhenius plots of different separators. i) Chronoamperometric curves of the symmetric batteries with different separators.

As illustrated in Figure S1 (Supporting Information), the sulfonated porous organic polymer (SPOP‐H) was synthesized by a solvothermal reaction of 2,4,6‐trihydroxybenzene‐1,3,5‐tricarbaldehyde and 4,4‐diamino‐2,2‐stilbenedisulfonic acid using a typical Schiff‐based process.^[^ ^25^ ^]^ Subsequently, SPOP‐H powder was reacted with LiOAc to swap their protons for Li^+^, culminating in dark red powder of SPOP‐Li nanofibers (Figure S2, Supporting Information).^[^ ^25^ ^]^ Fourier transform infrared (FT‐IR) spectra of SPOP‐H and SPOP‐Li show the characteristic peaks at 1279 and 1587 for C—N and C=C stretching vibration bands, respectively, confirming the establishment of keto‐enamine linkage (**Figure** **2**a).^[^ ^26^ ^]^ The peaks at 1025 and 1080 cm^−1^ can be assigned to the stretching of S=O, suggesting the existence of sulfonic groups. The newly formed O–Li band at 1617 cm^−1^ in the FT‐IR spectra demonstrates that the Li^+^ is coupled with the O atom in the SPOP‐Li backbone. The Li^+^ hopping paths are provided by the continuous ‐SO_3_Li sites in aligned nanochannels, which are based on the cation–dipole interaction.^[^ ^20^ ^]^ The specific surface area of SPOP‐Li is 279 m^2^ g^−1^ and the pore size is ≈1.3 nm characterized by the Brunauer–Emmett–Teller (BET) method (Figure S3, Supporting Information), which also shows the microporous distribution. The surface charge property of carbon nanotubes (CNTs), SPOP‐H, and SPO‐Li is evaluated by the zeta potential test (Figure 2b). CNTs, SPOP‐H, and SPOP‐Li show negative values of −3.01, −4.78, and −12.0 mV, respectively. The more negative property of SPOP‐Li is attributed to the presence of lithiated‐sulfonated groups, which will repel the PSs anions by electrostatic repulsion. Electrostatic potential distributions of SPOP‐Li exhibit a strong electron density in sulfonated groups region (blue color), which easily forms nucleophilic effect on LiPSs (Figure 2c).^[^ ^27^ ^]^ Namely, SPOP‐Li can block polysulfides by generating an electrostatic repulsion toward PSs anions and adsorbing molecules LiPSs. Meanwhile, the negatively charged sulfonated groups serve as abundant hopping sites for positively charged Li^+^, enhancing the Li^+^ conduction. Figure S5 (Supporting Information) shows the X‐ray diffraction (XRD) patterns of the LLZNO, indicating the good garnet crystalline structure.

The asymmetrical modified separator with dual coating layers (SPOP‐Li@PP@LLZNO) was manufactured using an industrially suitable blade coating process. The PP separator with nanometer‐sized slit pores (Figure S6, Supporting Information) is uniformly covered by SPOP‐Li and LLZNO layers (Figure 2d,e). The thicknesses of the SPOP‐Li and LLZNO layers are about 5 and 8 µm, respectively, which can be observed in the cross‐sectional SEM image (Figure 2f). The low loading of 0.12 and 0.25 mg cm^−2^ for SPOP‐Li and LLZNO, respectively, will not burden the mass energy density of the battery. Also, the energy‐dispersive X‐ray spectroscopy (EDS) reveals that SPOP‐Li and LLZNO are homogeneously coated on both surfaces of PP separator, respectively (Figures S7 and S8, Supporting Information). The formation of these two dense layers not merely blocks the migration of LiPSs, but also accelerates the transfer of Li^+^.

Some considerable important physicochemical parameters of the modified separator absorbed in liquid electrolyte, including ionic conductivity and Li ionic transference number (*t*_Li_^+^), were assessed.^[^ ^28^ ^]^ The pristine PP and CNTs coated PP (C‐PP) were used for comparison. From the electrochemical impedance spectra (EIS) (Figure S9, Supporting Information), the pristine PP and C‐PP present the ionic conductivity of 0.69 and 1.03 mS cm^−1^ at 25 °C, respectively. However, due to the superb Li^+^ transport channels in SPOP‐Li and LLZNO, both layers can facilitate the conduction of Li^+^ thus obtaining higher ionic conductivity of 1.24 mS cm^−1^. The Asy‐PP exhibits good Li^+^ conductivity at temperatures up to 105 °C (Table S1, Supporting Information), demonstrating its potential for operation in extreme environments. Figure 2h shows the Arrhenius plots acquired by fitting the ionic conductivity dependency on an ambient temperature of 25 to 105 °C. When the temperature rises to 75 °C, the ionic conductivity dependence with PP collapses. The dependency for Asy‐PP can be well fitted over a wide temperature range, and lower activation energy of 5.47 kJ mol^−1^ implies that Li^+^ cations are easier to transfer. Furthermore, the *t*_Li_^+^ was measured to demonstrate the single Li^+^ conduction behavior of Asy‐PP by a potentiostatic polarization method with Li/Li symmetric batteries (Figure 2i; Figure S10 and Table S2, Supporting Information).^[^ ^29^ ^]^ The Asy‐PP shows a superior *t*_Li_^+^ value of 0.86, which is remarkably higher than those of pristine PP (0.34) and C‐PP (0.49), respectively. The excellent results in Asy‐PP can be attributed to two factors: 1) lithiated‐sulfonated groups of microporous SPOP‐Li with electronegativity can facilitate electrolyte access, promote ion‐pair dissociation and increase Li^+^ mobility;^[^ ^30^ ^]^ 2) The LLZNO layer with abundant 3D Li^+^ transportation pathways can immobilize the anion and evenly redistribute the turbulences of Li^+^ flux distributions from the PP separator, contributing to a desirable homogenously Li plating behavior.^[^ ^21^, ^31^ ^]^

### 2.2 Polysulfides Blocking and Conversion Capability

**Figure 3:**
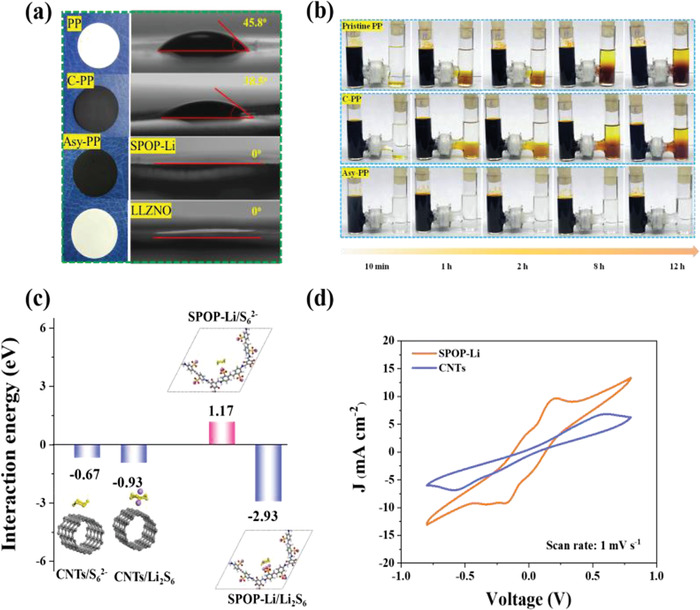
Polysulfides blocking and conversion capability. a) Photographs and contact‐angle images of PP, C‐PP, and Asy‐PP. b) Visual verification of the diffusion test of Li_2_S_6_ solution for PP, C‐PP, and Asy‐PP. c) Interaction between sulfur species (S_6_^2−^ and Li_2_S_6_) and various monomers. d) CV curves of symmetric cells with the electrodes of CNTs and SPOP‐Li in Li_2_S_6_ electrolyte.

Some measures are carried out to test the LiPSs blocking and conversion capability of the target materials. The contact angle test, **Figure** **3**a, showed the affinity between separators and liquid electrolyte. After 1 s infiltration, the contact angles are 45.8° and 38.5° for PP and C‐PP, respectively. In contrast, for Asy‐PP, the electrolyte immediately penetrated the SPOP‐Li and LLZNO layers, respectively, and exhibited a contact angle of 0°. The results are beneficial for enhanced ionic conductivity. The adsorption effect of different materials on LiPSs was displayed by adding 10 mg of samples to the Li_2_S_6_ solution (Figure S11, Supporting Information). After 12 h soaking, the CNTs and LLZNO‐containing solution showed slight discoloration, whereas the SPOP‐Li‐containing solution is nearly colorless. The LiPSs permeability through the different separators was carried out in an H‐shaped apparatus to better verify the effect of the Asy‐PP in preventing the LiPSs shuttling. As shown in Figure 3b, The Li_2_S_6_ solution quickly shuttles to the right chamber with the PP. Although the hysteresis of Li_2_S_6_ shuttling was observed with C‐PP after the same time, the physical barrier effect of CNTs on Li_2_S_6_ is unsatisfactory. In contrast, Asy‐PP can effectively inhibit LiPSs migration even after a long time of 12 h, showing that the SPOP‐Li acts as an ionic sieve and offers a superior LiPSs blocking effect.

The density functional theory (DFT) calculations were conducted to investigate the interaction energy of materials on polysulfides. Here, the representative polysulfides (S_6_^2−^ and Li_2_S_6_) were selected to investigate the interactions with SPOP‐Li and CNTs. As shown in Figure 3c, the interaction energies between CNTs and S_6_^2−^ as well as Li_2_S_6_ show negative values (−0.67 and −0.93 eV, respectively), indicating the binding behavior of CNTs toward PSs anions and LiPSs. While SPOP‐Li produces a larger negative value (−2.93 eV) with Li_2_S_6_, exhibiting a stronger binding interaction, which is mainly caused by the Lewis acid–base interaction between the electron‐rich sulfonic acid group and the electron‐deficient Li in Li_2_S_6_.^[^ ^32^ ^]^ The interaction energy between SPOP‐Li and S_6_^2−^ is positive (1.17 eV), indicating the presence of electrostatic repulsion between SPOP‐Li and PSs anions. From the above analysis, it is clear that the SPOP‐Li layer can block polysulfides by both electrostatic repulsion (i.e., repulsion of PSs anion) and trapping (i.e., adsorption of molecular LiPSs). The CNTs and SPOP‐Li were loaded on carbon paper (CP) as electrodes of the symmetrical cell and assembled with 0.2 M Li_2_S_6_ based electrolyte to investigate the LiPSs redox kinetics. As shown in Figure 3d, the cyclic voltammetric (CV) curves for the cell with SPOP‐Li exhibit obvious redox currents and less polarization than CNTs, suggesting that SPOP‐Li offers abundant redox sites and dynamically enhances LiPSs conversion kinetics, which will be confirmed by the discussion of Tafel plots in the section on electrochemical evaluation.

### 2.3 Homogeneous Li Deposition with Li–Mg Alloy Anode

**Figure 4:**
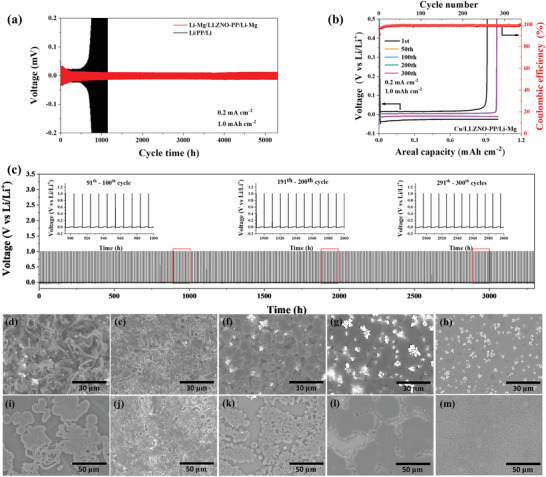
Li stripping/plating behavior. a) Cycling stability of Li/PP/Li symmetrical cells and Li–Mg/LLZNO‐PP/Li–Mg symmetrical cells at the current density of 0.2 mA cm^−2^ (1 mAh cm^−2^). b) Cycling CE and selected voltage profiles of Li–Mg/Cu half‐cells with LLZNO‐PP at the current density of 0.2 mA cm^−2^ (1 mAh cm^−2^). c) Li plating/stripping profile of the Li–Mg/LLZNO‐PP/Cu cell cycled at 0.2 mA cm^−2^ (1 mAh cm^−2^). Inset: selected cycles of charge−discharge curves of the Li–Mg/LLZNO‐PP/Cu cell from the bottom plot. SEM images of Li plating/stripping on the surface of Cu and Li (Li–Mg) electrodes. The first plating on the Cu electrode of the d) Li/PP/Cu, e) Li/C‐PP/Cu, f) Li–Mg/PP/Cu, g) Li–Mg/C‐PP/Cu, and h) Li–Mg/LLZNO‐PP/Cu cells, respectively. The first plating on the Li or Li–Mg electrode (corresponds to the first stripping from the Cu electrode) of i) Li/PP/Cu, j) Li/C‐PP/Cu k) Li–Mg/PP/Cu, l) Li–Mg/C‐PP/Cu, and m) Li–Mg/LLZNO‐PP/Cu cells, respectively.

Highly lithiated Li–Mg alloy was evaluated as a stable anode to potentially realize 500 Wh kg^−1^ LSBs.^[^ ^1^, ^33^ ^]^ In this regard, Asy‐PP as an ionic sieve can block the LiPSs shuttling and give a homogeneous Li^+^ distribution. When Li–Mg alloy and Asy‐PP are combined, a stable and dendrite‐free anode has the potential to achieve. Herein, to reflect the role of LLZNO as an ion redistribution, a double‐sided LLZNO‐coated separator (LLZNO‐PP) and pristine PP were used in symmetrical Li–Mg/LLZNO‐PP/Li–Mg and Li/PP/Li cells for evaluating the Li stripping/platting performance at 25 °C, respectively. When the current density was fixed at a low current density of 0.2 mA cm^−2^ (1 mAh cm^−2^), the voltage–time curve is depicted in **Figure** **4**a. For the commercial PP‐electrolyte and Li metal system, the voltage hysteresis is as large as ≈60 mV, then diverged and exceeded 100 mV after 700 h of cycling, suggesting that the electrolyte was almost exhausted due to continuously broken and repaired the fragile SEI and the gradual accumulation of dead Li. In contrast, the cell of Li–Mg/LLZNO‐PP/Li–Mg behaves a stable and narrower voltage hysteresis (<13 mV) for over 5300 h (≈7.36 months), indicating superb interfacial stability and dendrite‐free anode. A similar trend can be observed in high current density of 0.5 and 1 mA cm^−2^ and the battery can still cycle steadily for 1800 and 550 h, respectively, accompanied by possessing a stable and low polarization behavior (≈20 mV at 0.5 mA cm^−2^ and ≈35 mV at 1 mA cm^−2^) (Figure S12, Supporting Information). Then, the cells were disassembled after 100 cycles of testing at 1 mA cm^−2^ (Figure S13, Supporting Information). Figure S13a,e (Supporting Information) shows the massive cracks and Li dendrites with a thickness of 110 µm in a Li/PP/Li cell that accelerated the cell failure. Although the conductive CNTs reduce the interfacial resistance, unsatisfactory morphology, and corrosion thickness (77 µm) is still observed (Figure S13b,f, Supporting Information). Notably, there is a greater improvement in the surface and corrosion layer of the anode after replacing the anode with Li–Mg alloy in Li–Mg/PP/Li–Mg and Li–Mg/C‐PP/Li–Mg (Figure S13c,d,h,i, Supporting Information). Remarkably, a smoother dendrite‐free alloy anode surface was observed using the LLZNO‐PP with the “ion redistributor” effect of LLZNO, while the thickness of the reaction layer is minimal (44 µm) (Figure S13e,j, Supporting Information). It is shown that the homogeneous Li^+^ flux distribution generated by LLZNO and the stable Li–Mg alloy anode are both indispensable for achieving a dendrite‐free metal anode.

The CE of Li/Cu half‐cells quantifies the irreversible consumption of Li metal during cycling due to the formation of SEI and dead Li, which has a direct impact on the cycle life of a practical cell.^[^ ^31^, ^34^ ^]^ From Figure 4b, the average CE of Li–Mg/LLZNO‐PP/Cu cell exhibits above 99% over 340 cycles at a current density of 0.2 mA cm^−2^ (1 mAh cm^−2^), except for the initial low CE due to the activation reaction between the liquid electrolyte or LLZNO and Li–Mg alloy anode. The selected voltage profiles demonstrate the highly reversible Li stripping and plating, and the detailed charge and discharge curves of the 50th, 100th, 200th, and 300th render stable cycling and low polarization with a high CE. Figure 4c and the inset show more details on the Li stripping/plating cycling curves among 3300 h (≈4.6 months). In all stages of the cycling process, the Li plating/stripping curves display similar electrochemical behaviors, suggesting a stable interfacial structure after the formation of SEI and superb reversibility of cycling. In comparison, cells with PP and Li show a sharp CE decay after 85 cycles and remain a larger overpotential than that in Li–Mg/LLZNO‐PP/Cu cells in mere below 100 cycles (Figure S14, Supporting Information). The CE of the cell at higher currents of 0.5 and 1 mA cm^−2^ is also characterized, respectively. Li–Mg/LLZNO‐PP/Cu has better cycling stability and reversibility, once again demonstrating the superiority of this combination approach (Figure S15, Supporting Information).

The Li/Cu (Li–Mg/Cu) cells are disassembled to collect the electrodes for examination by SEM after the first Li plating and stripping, respectively. After the first Li plating on Cu, the Cu surface shows massive Li dendrites compared to the original Cu surface in Li/PP/Cu and Li/C‐PP/Cu cell (Figure 4d,e; Figure S16, Supporting Information), respectively. The Cu surface using the Li–Mg/Cu cell does not show long dendritic lithium, but particulate Li, which is caused by uneven Li^+^ flux from PP separator (Figure 4f). Since CNTs are beneficial for electron conduction rather than Li^+^ conduction, the surface of Cu still exhibits particulate Li without obvious improvement (Figure 4g). In contrast, a homogeneous Li deposition on the Cu surface is observed with almost no dendrites present in the Li–Mg/LLZNO‐PP/Cu cell (Figure 4h). Notably, the stripped Li surfaces appear to be very rough, while the Li–Mg surface delivers a porous and continuous morphology without much trace compared to the original Li–Mg surface (Figures S17 and S18, Supporting Information). When Li is stripped from Cu and deposited on top of Li metal, the SEM images were given in Figure 4i–m. In Figure 4i, regional nodule‐like structures with rounded edges were found on the Li surface from Li/PP/Cu cell. In Li/C‐PP/Cu cell, the presence of dendritic lithium can still be observed, forming an inhomogeneous deposit layer (Figure 4j). While comparing Li–Mg/PP/Cu, Li–Mg/C‐PP/Cu, and Li–Mg/LLZNO‐PP/Cu, the role of LLZNO as “ion redistribution” is revealed, and the Li–Mg surface shows dense Li deposition (Figure 4k–m).

### 2.4 Electrochemical Evaluation of LSBs

**Figure 5:**
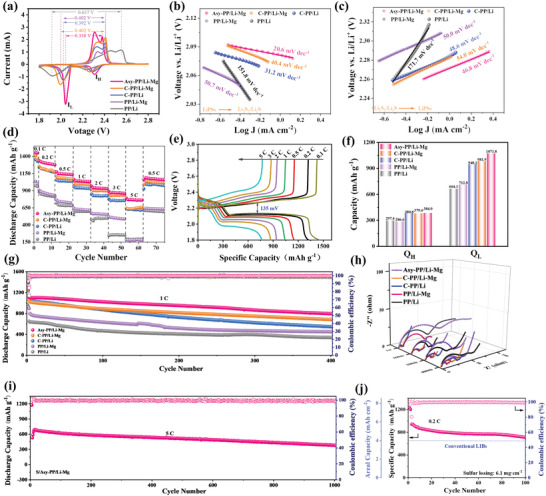
Electrochemical evaluation of LSBs. a) CV curves of different LSBs. b,c) The derived Tafel plots derived from the CV curves of the Li_2_Sn conversion reactions. d) Rate behavior and e) the corresponding charge/discharge curves. f) Capacity values for two discharge stages of *Q*_H_ and *Q*_L_. g) Cycling performance of different LSBs at 1 C (25 °C). h) EIS of the LSBs in various cycles at 1.0 C. i) Long cycling performance of the S/Asy‐PP/Li–Mg cell at 5 C. j) Cycling performances of the S/Asy‐PP/Li–Mg cell with high sulfur loading.

S/GO composite cathode (75%) with an area sulfur loading of 1.0–1.3 mg cm^−2^ was used to systematically investigate the electrochemical performance. The cells were in sequence denoted as the Asy‐PP/Li–Mg, C‐PP/Li–Mg, C‐PP/Li, PP/Li–Mg, and PP/Li. **Figure** **5**a shows the CV curves of all batteries from 1.8–2.8 V at 0.1 mV s^−1^. In the cathodic scan stage, two peaks at 2.3 and 2.0 V were observed, respectively, ascribing to the reduction reactions of S_8_ to soluble high‐order LiPSs (Li_2_S_x_, x = 4–8) and further conversion to Li_2_S_2_/Li_2_S. For the anodic scan, the peak at ≈2.4 V presents the reverse process of Li_2_S_2_/Li_2_S to S_8_.^[^ ^35^ ^]^ The Asy‐PP/Li–Mg cell shows sharper peaks and a narrower voltage polarization (0.338 V) than that in the C‐PP/Li–Mg (0.402 V), C‐PP/Li (0.392 V), PP/Li–Mg (0.402 V), and PP/Li (0.637 V) cell between the main reduction and oxidation peaks. Further, compared to the other batteries, the Asy‐PP/Li–Mg cell possesses a better overlap for the initial four circles and the highest collection coefficient (*I*_L_ */I*_H_) (Figure S19 and Table S3, Supporting Information), indicating the excellent electrochemical stability and fast kinetics of the sulfur redox reaction.^[^ ^36^ ^]^ The CV curves of different batteries under the scan rates from 0.1 to 0.5 mV s^−1^ were measured to evaluate the Li^+^ diffusion coefficient (*D*_Li_^+^). The peak currents value (*I*_p_) of A (reduction peaks), B and C (oxidation peaks) have a linear correlation with the square root of the scan (*V* ^0.5^) based on the Randles–Sevick equation.^[^ ^37^ ^]^ The slope of the fitted curves, which positively reflects the *D*_Li_^+^, was given in Figure S19 and Table S4 (Supporting Information). The Asy‐PP/Li–Mg cell has higher slope values of A, B, and C than those in others, respectively, indicating the stimulative Li^+^ migration and the enhanced redox reactions of LiPSs in Asy‐PP/Li–Mg cell. Subsequently, the Tafel plots of both oxidation and reduction peaks were derived for the evaluation of the electrocatalytic activities toward LiPSs conversion. From Figure 5b,c and Figure S20 (Supporting Information), the calculated slope values of Tafel plots in Asy‐PP/Li–Mg (20.6, 43.42, and 46.79 mV dec^−1^) are smaller than those in C‐PP/Li–Mg (40.4, 45.0, and 54.0 mV dec^−1^), C‐PP/Li cell (31.2, 49.3, and 48.6 mV dec^−1^), PP/Li–Mg cell (50.7, 65.4, and 50.0 mV dec^−1^), and PP/Li cell (151.8, 80.5, and 171.7 mV dec^−1^), suggesting SPOP‐Li in Asy‐PP/Li–Mg cell has considerable electrocatalysis toward the redox reaction of LiPSs conversion, which can be associated to the rapid reaction kinetics by Asy‐PP.

The rate behavior of various LSBs is compared in Figure 5d. The superior rate performance was performed in Asy‐PP/Li–Mg cell, which delivers a specific capacity of 1456, 1323, 1150, 1044, 941.7, 869.2, and 769.5 mAh g^−1^ at 0.1, 0.2, 0.5, 1, 2, 3, and 5 C (1 C = 1675 mA g^−1^), respectively. Once returned to 0.5 C, a reversible capacity of 1090 mA g^−1^ can be retrieved, which is ≈94.8% of the initial discharge capacity at 0.5 C (1150 mA g^−1^). The corresponding operational discharging–charging curves of Asy‐PP/Li–Mg cell display typical two‐plateaus even at a high rate of 5 C, while the load curves in other cells hardly show the stable reaction plateaus at 3 C or higher (Figure S21, Supporting Information). The comparison of the first charge–discharge curves of different LSBs at 0.1 C is depicted in Figure S22 (Supporting Information). The Asy‐PP/Li–Mg cell produces a voltage polarization of 135 mV at 0.1 C, whereas C‐PP/Li–Mg, C‐PP/Li, PP/Li–Mg, and PP/Li cell produce voltage polarizations of 138, 139, 140, and 139 mV, respectively, indicating that the cell with Asy‐PP/Li–Mg has better reaction kinetics. Figure 5f summarizes the calculation of two discharge plateau capacities, which present the redox reaction of the conversion from S_8_ to LiPSs (*Q*_H_) and LiPSs to Li_2_S_2_/Li_2_S (*Q*_L_), respectively. The Asy‐PP/Li–Mg cell exhibits a capacity of 384.0 and 1072 mA g^−1^ for *Q*_H_ and *Q*_L_, respectively, higher than those in C‐PP/Li–Mg (379.6 and 981.9 mA g^−1^), C‐PP/Li (380.8 and 948.1 mA g^−1^), PP/Li–Mg (286.6 and 712.8 mA g^−1^), and PP/Li (297.4 and 664.1 mA g^−1^) cells.

As shown in Figure S23 (Supporting Information), after the initial two cycles for S activation at 0.1 C, Asy‐PP/Li–Mg cell delivers an initial discharge capacity of 1181 mA g^−1^ and a slow decease to 875.5 mAh g^−1^ with a decay rate of 0.12% per cycle at 0.5 C. By comparison, the other cells show relatively low discharge capacity and rapid capacity degradation rate, which are inferior to the cycling stability of Asy‐PP/Li–Mg cell. When the current density maintained at 1 C (Figure 5g), the C‐PP/Li–Mg, C‐PP/Li, PP/Li–Mg, and PP/Li cells deliver a lower discharge capacity of 1059, 1094, 894.6, and 651.3 mA g^−1^, and drops dramatically to 683.0, 540.7, 450.2, and 340.5 mA g^−1^ after 400 cycles, respectively. Surprisingly, the Asy‐PP/Li–Mg cell delivers a higher initial capacity of 1116 mA g^−1^ and slow decay to 794.5 mA g^−1^ with an average CE of 99.5%. The discharge capacity degradation rate is merely 0.07% per cycle at 1 C, showing the high utilization rate and stable Li–Mg alloy anode in the Asy‐PP/Li–Mg cell. Figure 5h depicts the EIS tests of different LSBs before and after various cycles at 1 C for further investigation of the reaction kinetics. The C‐PP/Li–Mg and C‐PP/Li cell show a lower charge‐transfer resistance before cycling because of the reduced interface resistance by CNTs layers, respectively. After cycling, the resistance of all cells drops dramatically compared to the pre‐cycling, which is caused by the dissolution and redistribution of insulating S during chemical activation (Figure S24, Supporting Information). The battery exhibits minimal resistance after 400 cycles for Asy‐PP/Li–Mg cell, which indicates the effective inhibition of LiPSs and enhanced reaction kinetics.

The long‐term cycling stability of LSBs at high current densities was also explored, which is critical for improving the charging speed. Herein, the Asy‐PP/Li–Mg cell was tested for ultra‐long cyclability at 2, 3, and 5 C, respectively (Figure 5i; Figure S25, Supporting Information). The cell delivers an ultrahigh discharge capacity of 1020 mA g^−1^ with a CE of over 99.0% and remains 664.8 mA g^−1^ after 500 cycles at 2 C. A similar superior performance at 3 C is also observed. The initial discharge capacity is 841.4 mA g^−1^ at 3 C and a low degradation rate of ≈0.06% is also obtained. Notably, after the initial two cycles of activation at 0.1 C, the Asy‐PP/Li–Mg cell provides a low initial capacity of 538.5 mAh g^−1^ and attains a maximum capacity of 692.3 mAh g^−1^ after 10 cycles at 5 C (Figure 5i). This is because the sulfur cathode necessitates an activation stage at high current densities that engage more active S in the electrochemical process. After 1000 cycles, the decay rate per cycle was calculated to be 0.03% and high CE was maintained throughout the cycling (>99%), indicating high sulfur utilization and battery stability at a large current density.

Highly loaded sulfur cathodes are the key to achieve the practical application of high‐energy‐density LSBs. The aqueous binder‐based highly sulfur loading (6.1 mg cm^−2^) is constructed and applied in Asy‐PP/Li–Mg cell (Figure 5j).^[^ ^38^ ^]^ The assembled Asy‐PP/Li–Mg cell shows a high initial specific capacity of 943 mAh g^−1^ and the corresponding areal capacity is 5.75 mAh cm^−2^ at 0.2 C. Furthermore, a desired high discharge and areal capacities of 711.7 mAh g^−1^ and 4.34 mAh cm^−2^ are still maintained after 100 cycles, respectively, better than the state‐of‐the‐art commercial LIBs. The selected galvanostatic discharge–charge curves are given in Figure S26 (Supporting Information), the characteristic plateaus and small voltage polarization can be observed, suggesting excellent stability and reversibility of the battery. These results indicate that Asy‐PP/Li–Mg cell has promising potential for the commercial viability of LSBs.

**Figure 6:**
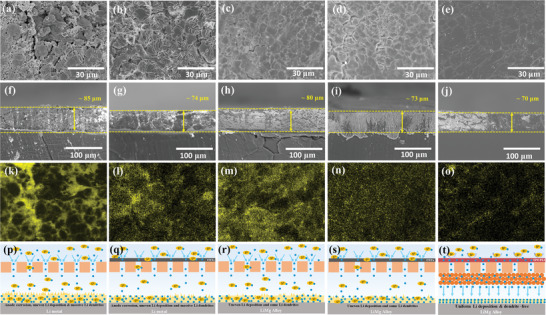
Morphological analysis of LSBs after cycling. SEM images of a–e) top‐view and f–j) cross‐section of cycled Li (Li–Mg) anode, and the corresponding k–o) surface of EDS analysis, from the a,f,k) PP/Li, b,g,l) C‐PP/Li, c,h,m) PP/Li–Mg, d,i,n) C‐PP/Li–Mg, and e,j,o) Asy‐PP/Li–Mg cell, respectively. p–t) Schematic illustration of the cyclic process in the p) PP/Li, q) C‐PP/Li, r) PP/Li–Mg, s) C‐PP/Li–Mg, and t) Asy‐PP/Li–Mg cell, respectively.

The different LSBs were disassembled after 100 cycles to analyze the effect on the anode. From **Figure** **6**a, the Li surface from the Li/PP cell shows serve cracks and porous structure. Similarly, the undesirable surface situation of Li anode from C‐Li/PP is observed in Figure 6b, showing unevenness and massive dendrite growth. In contrast, the Li–Mg alloy anode shows a smooth and dendrite‐free anode surface from the PP/Li–Mg, C‐PP/Li–Mg, and Asy‐PP/Li–Mg cell, respectively (Figure 6c–e). Especially, the anode surface of Asy‐PP/Li–Mg cell is dense and immaculate, which can be mainly attributed to two reasons. First, Asy‐PP prevents the erosion of the anode by LiPSs and produces a homogeneous Li^+^ flux distribution. On the other hand, the Li–Mg alloy retains the stability of the anode bulk during the Li stripping/plating process, i.e., the Li stripping produces an ion‐electron co‐conductive framework.^[^ ^39^ ^]^ This framework reduces the local current density, directing subsequent lithium deposition on such a porous substrate, thereby limiting the volume change. The corresponding S mapping was also detected in Figure 6k–o. The Li–Mg anode of the Asy‐PP/Li–Mg cell shows the lightest color compared to the other cells, which proves that the SPOP‐Li modified layer has a good ability to block LiPSs and accelerated LiPSs conversion. Besides, the reaction depth of the anode can be observed from the cross‐section SEM images (Figure 6f–j). Thickness of 85, 74, 80, 73, and 70 µm was obtained for PP/Li, C‐PP/Li, PP/Li–Mg, C‐PP/Li–Mg, and Asy‐PP/Li–Mg cell, respectively. The fact is clear that the crossed LiPSs interact with the anode are eventually reduced to Li_2_S_2_/Li_2_S on the anode surface.^[^ ^40^ ^]^ Moreover, the pure Li anode is more prone to volume expansion and pulverization compared to Li–Mg alloy anode.^[^ ^24^ ^]^ These two reasons account for the increased thickness. As illustrated in Figure 6p, the PP separator without inhibiting of LiPSs shuttling makes the Li anode passivation and erosion. And then, because of the inert Li^+^ conductivity of the commercial PP separator, ions are concentrated in the surrounding pores and leading to an exceedingly nonuniform Li^+^ distribution after the PP separator. Further, the unstable Li anode intensifies the growth of Li dendrites, which eventually leads to the generation of “dead Li” over extended cycling. Despite some changes have done in the C‐PP/Li, PP/Li–Mg, and C‐PP/Li–Mg cell, including CNTs modified separator and altered anode material, respectively (Figure 6q–s), the insufficient polysulfide limitation, uneven Li^+^ deposition and unsuitable Li anode still corrode the anode surface. In Asy‐PP/Li–Mg cell (Figure 6t), the Asy‐PP with SPOP‐Li and LLZNO has the significant electronegative capacity to block LiPSs and delivers a homogeneous Li^+^ distribution combined with a stable Li–Mg alloy anode, yielding a stable SEI layer and a dendrite‐free anode.

## 3 Conclusion

In summary, we designed a versatile asymmetric separator and a Li‐rich Li–Mg alloy anode for LSBs. The asymmetric separator was created by coating a PP separator with the SPOP‐Li layer and the LLZNO layer on two sides to address the dissimilar interface concerns. The SPOP‐Li with the concentrated negative sulfonated groups could suppress the LiPSs shuttling by both electrostatic repulsions of PSs anions and adsorption of molecular LiPSs. Simultaneously, SPOP‐Li with lithiated‐sulfonated groups could serve as Li conductors for accelerating Li^+^ migration. On the other side, LLNZO acts as a 3D Li^+^ conductor, guiding a uniform Li^+^ flux on the anode. Combined with the exceptionally stable Li–Mg alloy, a dendrite‐free anode and high‐performance can be obtained for LSBs. Hence, the LSBs with Asy‐PP/Li–Mg exhibit a high initial discharge capacity of 1116 mA g^−1^ at 1 C and cycle for 400 cycles with CE of ≈99.5%. At a high current density of 5 C, an extraordinarily low attenuation rate (0.03%) per cycle for 1000 cycles is attained. Besides, a desirable reversible areal capacity is achieved with a high S cathode loading. This work provides novel insights into the development of commercially available of LSBs with high‐energy density.

## Conflict of Interest

The authors declare no conflict of interest.

## Supporting information

Supporting Information

## Data Availability

The data that support the findings of this study are available from the corresponding author upon reasonable request.
