# Supplementary material for: Versatile Asymmetric Separator with Dendrite‐Free Alloy Anode Enables High‐Performance Li–S Batteries
Source: Adv Sci (Weinh). 2022 Jun 24;9(25):2202204. doi: 10.1002/advs.202202204 (PMC9443453; doi:10.1002/advs.202202204)
Supplement: Supplementary file 1 — Supporting Information [file ADVS-9-2202204-s001.pdf]

ADVANCED SCIENCE
Open Access

Supporting Information

for *Adv. Sci.*, DOI 10.1002/advs.202202204

Versatile Asymmetric Separator with Dendrite-Free Alloy Anode Enables High-Performance Li–S Batteries

Wenqi Yan, Jin-Lin Yang, Xiaosong Xiong, Lijun Fu, Yuhui Chen, Zhaogen Wang, Yusong Zhu, Jian-Wei Zhao, Tao Wang and Yuping Wu**

Supporting Information

Versatile asymmetric separator with dendrite-free alloy anode enables high-performance Li-S batteries

Wenqi Yan, Jin-Lin Yang, Xiaosong Xiong, Lijun Fu, Yuhui Chen, Zhaogen Wang, Yusong Zhu, Jian-Wei Zhao, Tao Wang and Yuping Wu**

W.Q. Yan, X.S. Xiong, Prof. L.J. Fun, Y.H. Chen, Z.G Wang, Y.S. Zhu, Y.P. Wu
State Key Laboratory of Materials-oriented Chemical Engineering
Institute of Advanced Materials (IAM) and School of Energy Science and Engineering
Nanjing Tech University
Nanjing 211816, P.R. China
E-mail: wangzhaogen@njtech.edu.cn;
wuyp@njtech.edu.cn

J.L. Yang
School of Physical and Mathematical Sciences
Nanyang Technological University
Singapore 637371, Singapore

Tao Wang, Y.P. Wu
School of Energy and Environment,
Southeast University,
Nanjing 211189, P.R. China

J.-W. Zhao
Shenzhen HUASUAN Technology Co. Ltd
Shenzhen 518055, P.R. China

Materials

2,4,6-Trihydroxybenzene-1,3,5-tricarbaldehyde and 4,4-diamino-2,2-stilbenedisulfonic acid, mesitylene, 1,4-dioxane, acetic acid (HOAc) and lithium acetate (LiOAc) were purchased from TCI Reagent Co. $Li_{6.75}La_3Zr_{1.75}Nb_{0.25}O_{12}$ (LLZNO) nanoparticles were purchased from WuXi Kai-Star Electro-Optic Materials Co. Ltd. All of the chemicals are of analytical grade and were used without further purification.

Synthesis of SPOP-H and SPOP-Li

The SPOP-H was synthesized by a solvothermal reaction. Specifically, 2,4,6-Trihydroxybenzene-1,3,5-tricarbaldehyde (31.5mg, 0.15mmol) and 4,4-Diamino-2,2-stilbenedisulfonic acid (83.3mg, 0.225mol) were dispersed in a Pyrex tube containing with the solution of mesitylene (2.4mL)/ 1,4-dioxane (0.6mL)/ 3M HOAc (0.6ml), and the mixture was sonicated for 10 min. Immediately following the rapid freezing of the tube in liquid nitrogen (77K) and degassed by three freeze-pump-thaw cycles, and then flame sealed. Afterward, the tubes were placed in an oven at 120 °C for 3 days. Finally, the product was filtered and washed with water, ethanol and acetone. The final reaction product is collected by vacuum drying at 100 °C overnight (yield: 109 mg, 85.8%). The SPOP-Li (100 mg) was synthesized by suspending the above SPOS-H in a 5 M LiOAc solution and stirred for 3 days at room temperature. After washing with deionized water to remove the excess LiOAc, the SPOP-Li powers were collected after drying under vacuum at 100 °C overnight (yield: 88.5 mg, 88.5%). The illustration of the synthesis and chemical structure of SPOP-H and SPOP-Li was shown in Figure. S1.

Preparation of the asymmetric separator

The asymmetric separators are prepared by coating different layers on both sides. In detail, the obtain SPOP-Li material (70mg), carbon black (20mg) and polyethylene oxide (PEO, 10mg) were mixed in N-methyl-2-pyrrolidone (NMP) and stirred for 6 h to form a homogeneous slurry. Then, the homogeneous slurry was cast onto a PP separator (Celgard 2400) by a doctor blade, and dried at 60 °C overnight in a vacuum. The other

side of the pp separator is coated with LLZNO. Specifically, 90 mg of LLZNO and 10 mg of PEO were added in NMP and stirred for 6 h to form a homogeneous slurry. Then the dispersion slurry was coated on the other side of the above PP separator with SPOP-Li coating. After drying and then punched into 19 mm disks stored in Argon glovebox for further use. The carbon nanotubes (CNTs) coated PP separator is prepared regarding the above method.

Sulfur/GO cathode preparation

Commercial sublimed sulfur powders (S) and graphene oxide (GO) (3:1 w/w) were mixed by ball milling for 12h at 250rpm, followed by encapsulating S in GO under 155 °C argon atmosphere for 24 h. Subsequently, S/GO composite, PVDF, and carbon black (8:1:1 w/w/w) were mixed in NMP and stirred for 6 h to make a homogeneous slurry. The S/GO cathodes with a sulfur areal loading of 1.0-1.3 mg cm^{-2} were obtained by coating on the carbon-coated aluminum foil and vacuum drying for 12h. The high-loading S/GO cathodes (> 5.0 mg cm^{-2}) were prepared by using an aqueous-based binder of carboxymethylcellulose sodium (CMC) and styrene butadiene rubber (SBR) dispersion solution (48w%) instead of PVDF. Firstly, 22 mg of CMC was added in 2ml deionized water and vigorously stirred for 60 min to completely dissolve, and then 43 mg of carbon black was slowly added with stirring, followed by stirring for another 90 min. after that, 988 mg of S/GO active material was added and stirring was continued for 120 min. Finally, 56 mg of SBR was added and stirred for 30 min to obtain a homogeneous slurry. The same as the above procedure, the homogeneous slurry was coated on carbon-coated Al foil by using a doctor blade and dried in a vacuum oven at 60 °C overnight.

Materials Characterizations

The morphologies and element distribution mappings of materials were obtained by using scanning electron microscopy (SEM, JEOL JSM-7800F). The X-ray diffraction (XRD, SmartLab) of LLZNO was performed from 10-70 ° with Cu-Kα radiation (10° min^{-1}). Fourier-transform infrared (FTIR) spectra was measured by a BRUKER

ALPHA spectrometer (4000–400 cm^{-1}, resolution 2 cm^{-1}). The surface area and pore size were collected with Quantachrome autosorb IQ3 system. The surface chemical composition was carried out with X-ray photoelectron spectroscopy (XPS, VG Scientific ESCALAB 2201XL).

Electrochemical Characterizations

Li$^+$ ionic conductivity

The blocking stainless steel (SS)/separator-electrolyte/SS symmetric cells were assembled to conduct the electrochemical impedance spectroscopy (EIS) analysis (1-10^5 Hz, 10 mV) by the electrochemical station (CHI 760E, Chenhua) to obtain the impedance of the different separators from 25 to 105 °C. The ionic conductivity(σ) was calculated by the equation (S1):

$$\sigma = \frac{l}{R_b \times A} \text{ (S cm}^{-1}\text{)} \qquad \text{(S1)}$$

Where l and A are the thickness of the separator and the contacted area, respectively.

Li-ion transference number

The transference number of Li$^+$ was estimated by the chronoamperometry test. The symmetric Li/Li cells were assembled with PP separator or modified separator on an electrochemical working station. The initial resistance (R_0) was measured by electrochemical impedance spectroscopy (EIS). The initial current (I_0) and the steady-state current (I_s) can be acquired after 1000 s' chronoamperometric measurement with a constant potential step difference of 10 mV (ΔV). After the chronoamperometry measurement, the steady-state resistance (R_S) was measured Li-ion transference number (t_{Li^+}) can be calculated by the Equation (S2):

$$t_{Li^+} = \frac{I_S(\Delta V - I_0 R_0)}{I_0(\Delta V - I_S R_S)} \qquad \text{(S2)}$$

Cycling performance and Li plating/stripping behavior

S/GO cathodes, modified separators and lithium-magnesium alloy foil (90w% Li) anode was assembled using CR2025 type coin cells. The electrolyte using in the batteries is 1 M LiTFSI (DME/DOL, 1:1, v/v) with 2.0 % $LiNO_3$. The batteries for

comparison were assembled by replacing the separator and anode (Li metal). all processes of assembling batteries were carried out in an Ar-filled glovebox (H_2O < 0.1 ppm, O_2 < 0.1 ppm). The cycling and rate performance of batteries from 1.8 to 2.8 V was evaluated by the battery test system (LANHE CT2001A, Wuhan LAND Electronics Co). Cyclic voltametric (CV) curves, EIS (10^{-1}-10^{5} Hz) before and after cycling were characterized by the electrochemical station. The Li plating/stripping behavior was performed by Li/Li symmetric cells and Li/Cu cells with various separators under a current density of 0.2, 0.5 and 1mA cm^{-2} (1mAh cm^{-2}), respectively. For the Li/Cu cells, the cutoff voltage when the lithium metal is stripped from the Cu is 1 V. Because Li stripping/platting occurs on the metal anode. Therefore, to reflect the role of LLZNO as an ion redistribution, a double-sided LLZNO-coated separator (LLZNO-PP) was used in Li-Mg/Cu and Li-Mg/Li-Mg cells for evaluating the Li stripping/platting performance.

Li_2S_6 visualized adsorption and diffusion tests

Li_2S_6 solution was prepared by the reaction equation (S3):

$$5S + Li_2S \rightarrow Li_2S_6 \quad \text{(S3)}$$

Sulfur powder and Li_2S (AR, Aladdin) were dissolved in DOL/DME (1:1, v/v) solution in a ratio of 5:1 by stirring at 60 °C for 24h to obtain a 0.2 M Li_2S_6 solution. For the Li_2S_6 diffusion tests, the H-shaped apparatus with Li_2S_6 solution and pure DOL/DME solution was assembled with various separators to test the separators in blocking the LiPSs shutting effect. For the visualized adsorption test, 10 mg of CNTs, LLZNO and SPOP-Li were added to 2 mM Li_2S_6 solution and rested for different hours.

Li_2S_6 Symmetric Cells

The electrodes of Li_2S_6 symmetric cells were obtained by loading CNTs and SPOP-Li on carbon cloth (CC). The electrolyte is 0.2 M Li_2S_6 solution in the electrolyte (1 M LiTFSI in DOL/DME = 1/1 by volume). The symmetric cells were assembled with the above electrodes as working and counter electrodes with 40 μL of Li_2S_6 solution. CV tests were carried upon a voltage window from −0.8 to 0.8 V.

All tests are conducted at room temperature (25 °C) except for special notations.

Computational details

The density functional theory (DFT) calculations, which were implemented in the Vienna ab initio simulation package (VASP) code,[1]wasapplied to calculate the binding energies of S^{6-} and $Li^{2}S^{6}$ clusters. The projector augmented wave (PAW) method was applied to describe the electron-ion interaction.[2] And the electron exchange correlation was represented by the functional of Perdew, Burke and Ernzerhof (PBE) of generalized gradient approximation (GGA).[3] For all the calculations, the cutoff energy was set to be 500 eV. All periodic slab calculations were carried out using a vacuum spacing of at least 15 Å to avoid the interaction between the neighboring periodic structures. The convergence tolerance was 10^{-5} eV and 0.01 eV/Å for energy and force, respectively. The Brillouin zone integrations were performed by using 1×1×3 Monkhorst-Pack for carbon nanotube and 2×2×1 Monkhorst-Pack for SPOP-Li. In addition, DFT-D3 calculations were adopted to describe the van der Waals (vdW) interaction.[4] The electrostatic potential distribution of SPOP-Li was also obtained via VASP. Multiwfn loads the LOCPOT when LVHAR=.TRUE. And the cub file with negative values of electrostatic potential was exported with option 0 in main function 13. Then Multiwfn loads CHGCAR and the cub file for electron density was exported in the main function 13 with option 0. Finally, both files were loaded into the VMD, and the equivalence surface was displayed with the cub file of the electron density and colored according to the negative cub file of the electrostatic potential.

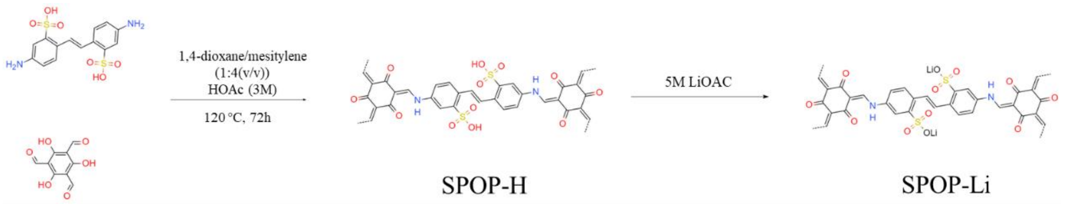

Figure S1. Schematic illustration of the synthesis and chemical structure of SPOP-H and SPOP-Li.

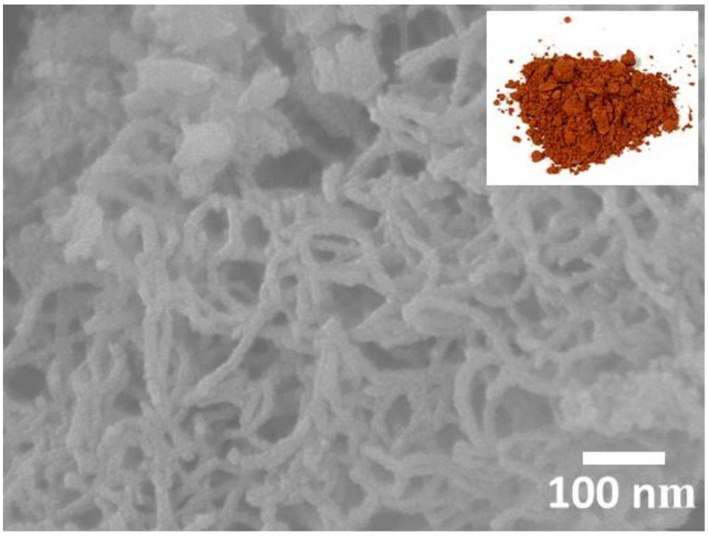

Figure S2. SEM images of the synthesized SPOP-Li, inset is the optical photos.

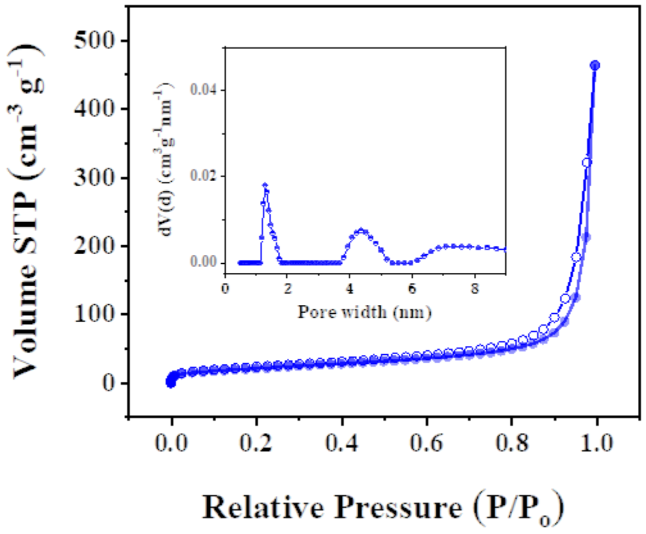

Figure S3. N_2 adsorption/desorption isotherms and the pore size distribution curve of SPOP-Li.

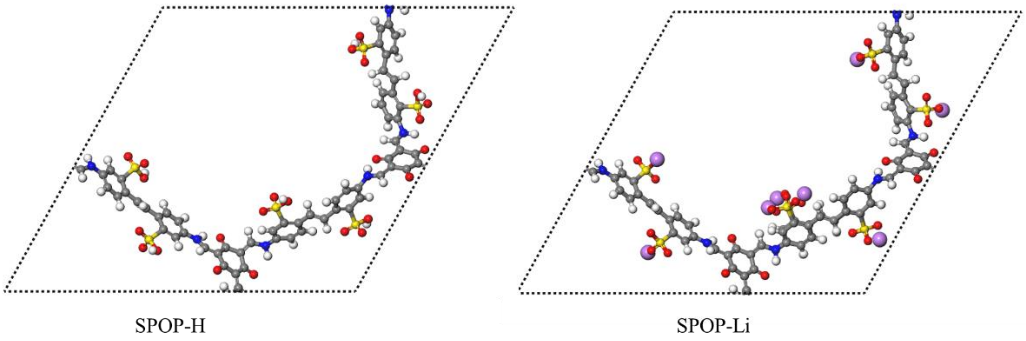

Figure S4. Structure of the synthesized SPOP-H and SPOP-Li (red, oxygen; blue, nitrogen; grey, carbon; white, Hydrogen; purple, lithium).

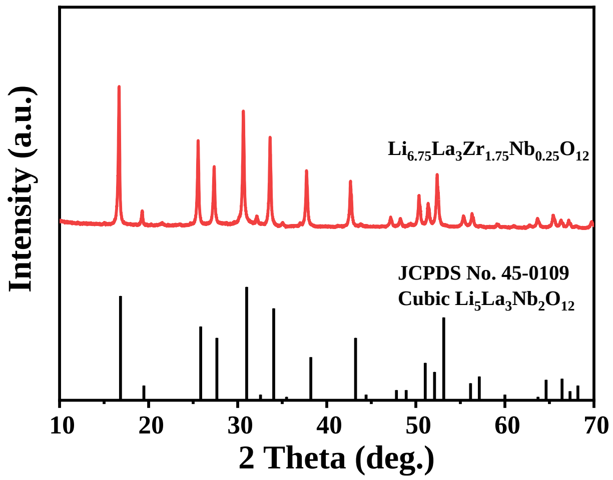

Figure S5.XRD of LLZNO.

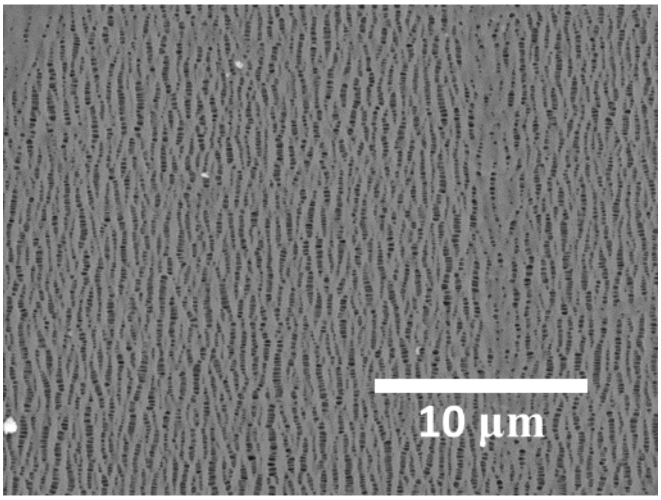

Figure S6. SEM images of the surface of PP separator.

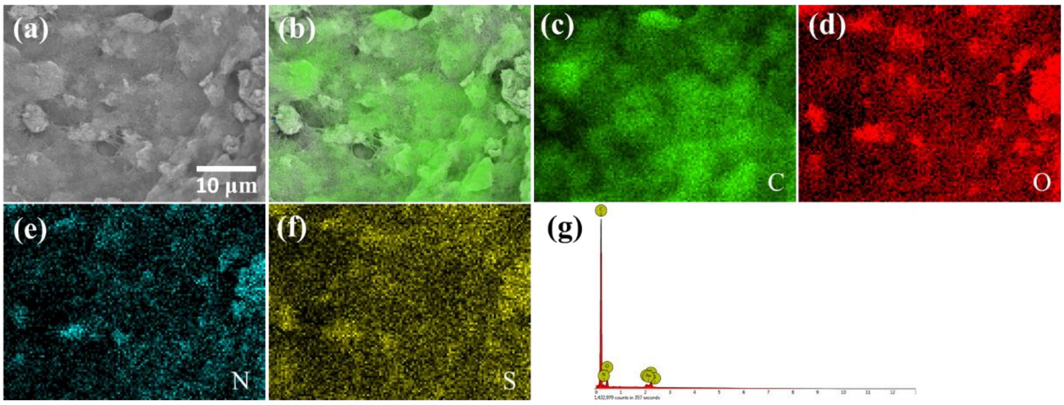

Figure S7. SEM images and EDS analysis of SPOP-Li layer.

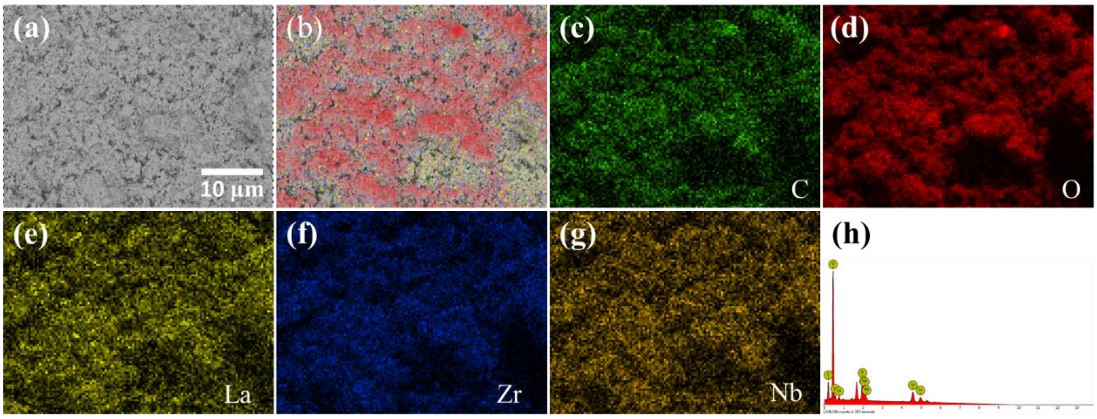

Figure S8. SEM images and EDS analysis of LLZNO layer.

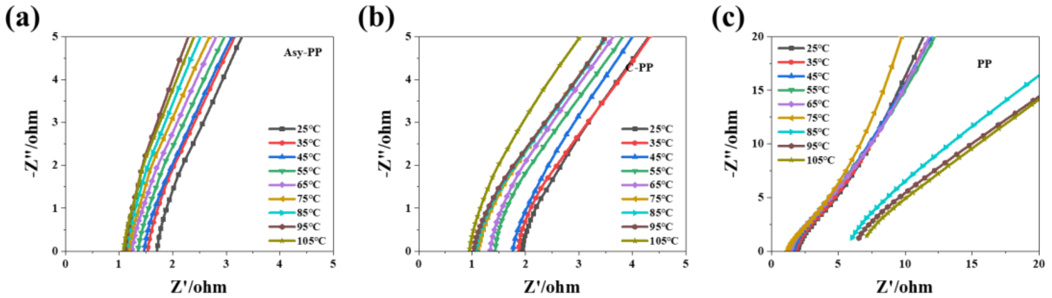

Figure S9. EIS plots of Asy-PP, C-PP and PP at different temperatures.

Table. S1 EIS data and the corresponding ionic conductivity of different separators for 25 to 105 °C.

	Pristine PP (25μm)		C-PP (33μm)		Asy-PP (38μm)	
Temperarture (°C)	R_b (Ω)	δ (mS cm^{-1})	R_b (Ω)	δ (mS cm^{-1})	R_b (Ω)	δ (mS cm^{-1})
25	1.958	0.69	1.928	1.03	1.687	1.24
35	1.777	0.74	1.854	1.06	1.5	1.40
45	1.581	0.83	1.758	1.14	1.433	1.46
55	1.43	0.92	1.418	1.39	1.322	1.58
65	1.342	0.98	1.32	1.48	1.205	1.74
75			1.094	1.81	1.152	1.82
85			1.046	1.88	1.109	1.89
95			1.005	2.96	1.088	1.92
105			0.9304	2.12	1.064	1.97

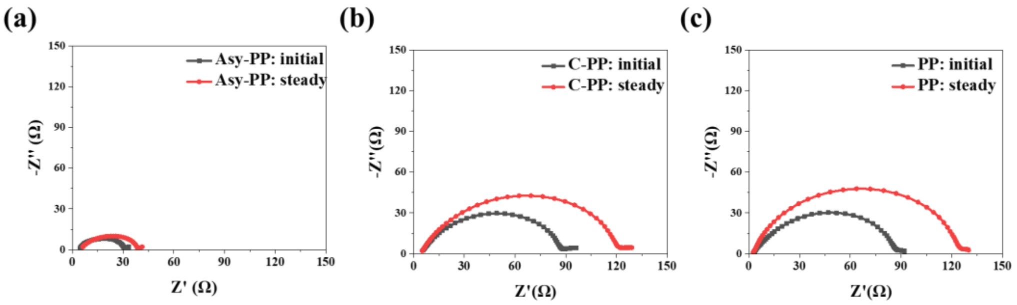

Figure S10. Nyquist plots before and after polarization.

Table S2. Parameters related to Li-ion transference number.

Types	I_0	I_s	R_0	R_S	t_{Li+}
Asy-PP	126.5	109.3	27.5	32.0	0.86
C-PP	160.5	78.3	62.1	121.8	0.49
PP	91.3	36.5	64.5	141.4	0.34

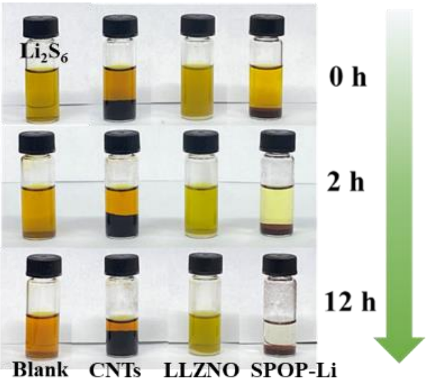

Figure S11. Photographs of the color changes of the Li_2S_6 solution exposed to different materials.

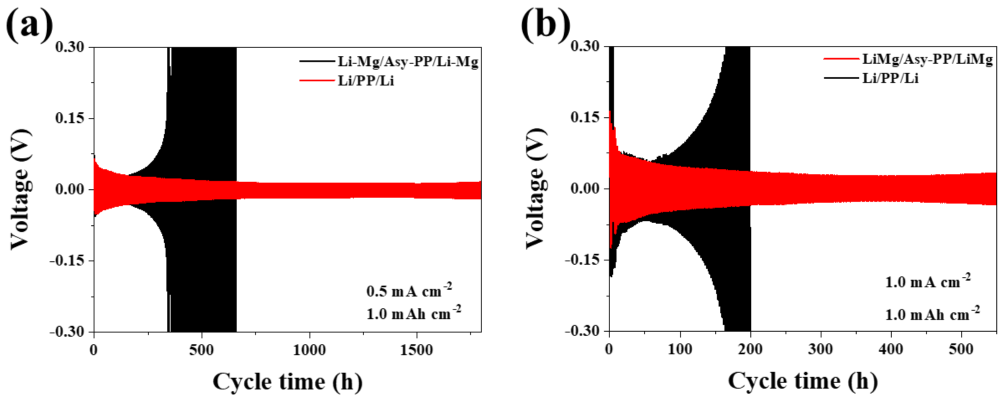

Figure S12 Cycling stability of Li-Mg/Li-Mg symmetrical cells with LLZNO-PP the current density of (a) 0.5 and (b) 1.0 mA cm^{-2} (1.0 mAh cm^{-2}).

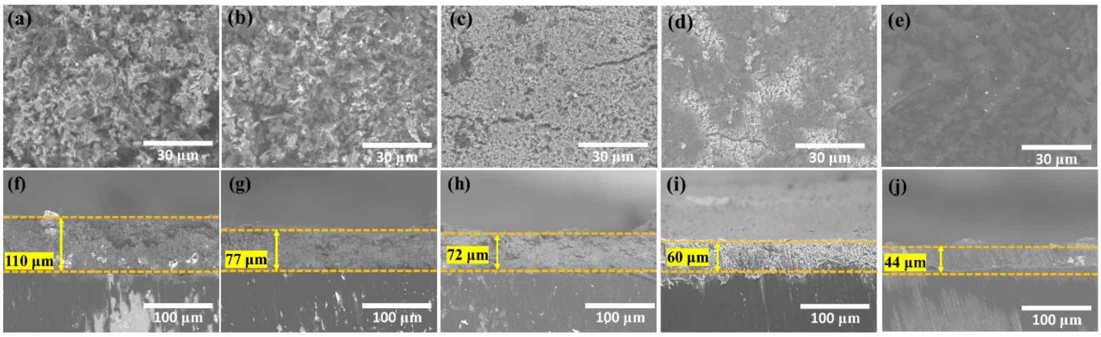

Figure S13 (a-e) Top-view and (f-j) cross-sectional SEM morphologies of the anodes after 100 cycles of testing at 1 mA cm^{-2} from a (a, e) Li/PP/Li cell, (b, f) Li/C-CNT/Li, (c, g) Li-Mg/PP/Li-Mg, (d, h) Li-Mg/C-PP/Li-Mg and (e, j) Li-Mg/LLZNO-PP/Li-Mg.

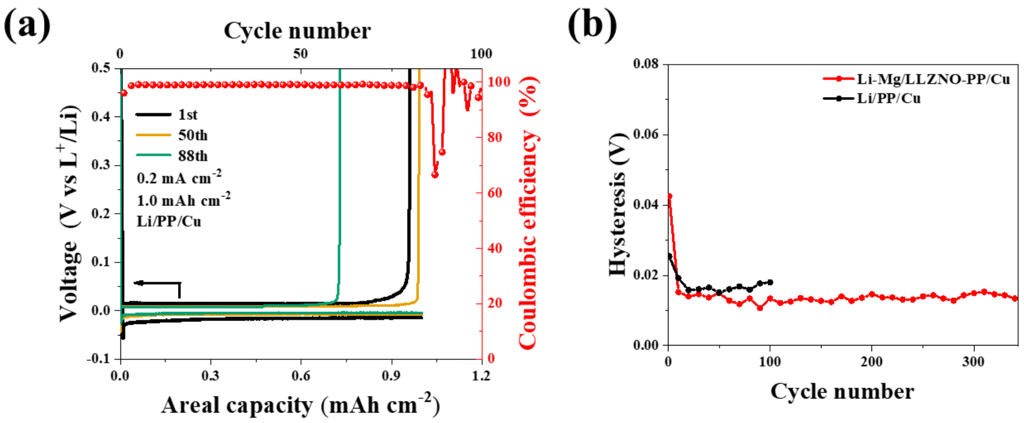

Figure S14 (a) Cycling CE and selected voltage profiles of Li/Cu half-cells with PP at the current density of 0.2 mA cm^{-2} (1 mAh cm^{-2}). (b) The Comparison of hysteresis voltage of Li/PP/Cu and Li-Mg/LLZNO-PP/Cu.

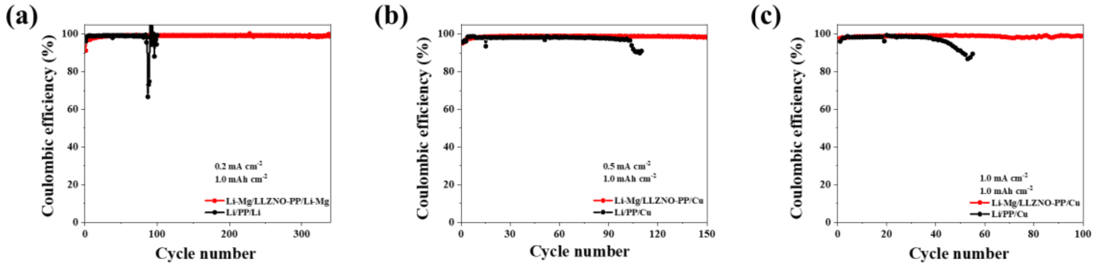

Figure S15 Comparison of CE at the current density of 0.2, 0.5 and 1 mA cm^{-2} (1 mAh cm^{-2}), respectively.

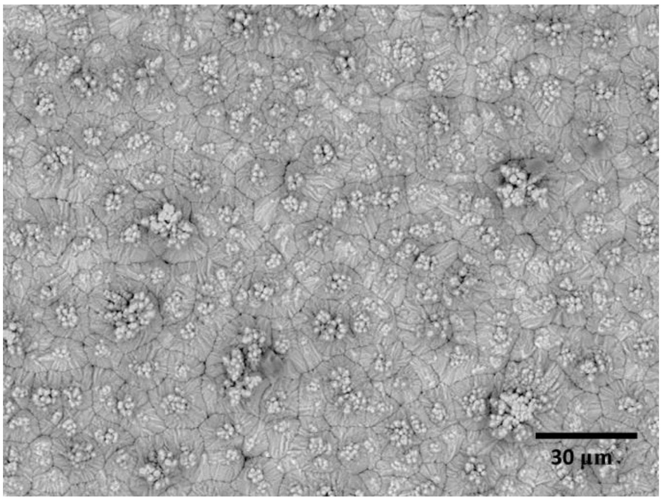

Figure S16 SEM images of the surface of pristine Cu.

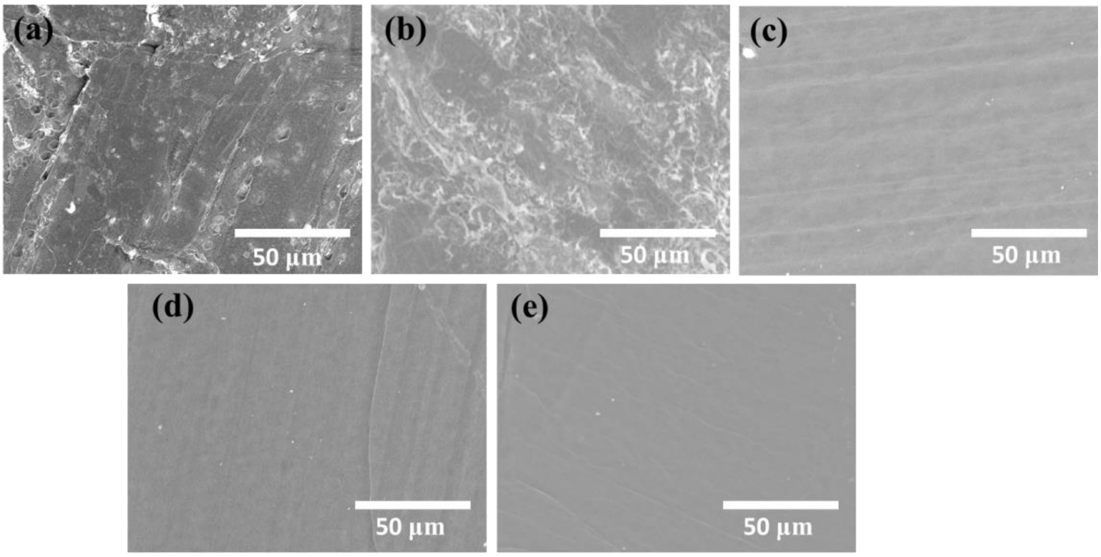

Figure S17 SEM images of the Li (Li-Mg) surface from (a) Li/PP/Cu cells, (b)Li/C-PP/Cu, (c) Li-Mg/PP/Cu, (d) Li-Mg/C-PP/Cu and (e) Li-Mg/LLZNO-PP/Cu cells after the first Li stripping from Li.

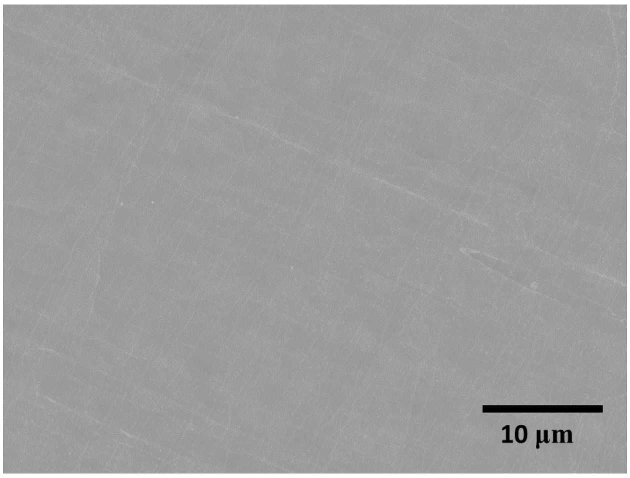

Figure S18 SEM images of the surface of pristine Li-Mg alloy.

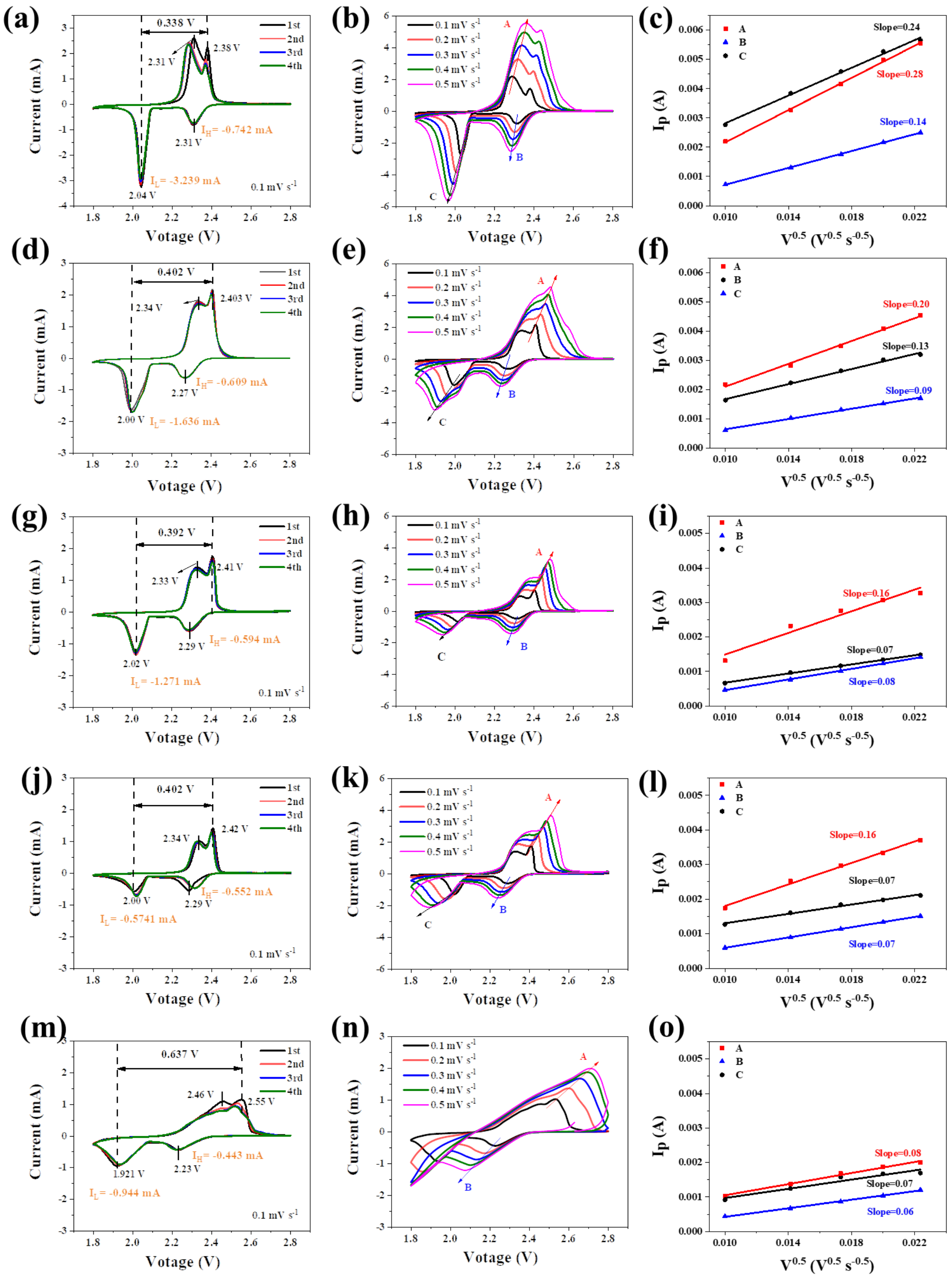

Figure S19. CV curves at various scan rates and the corresponding linear fits of the peak, for (a-c) Asy-PP/Li-Mg , (d-f) C-PP/Li-Mg, (g-i) C-PP/Li, (j-l) PP/Li-Mg and (m-o) PP/Li cell.

Table S3. The voltage polarization and collection coefficient (I_L/I_H) values derived from the CV profiles in **Figure S19**a, d, g, j and m.

Cell	Voltage polarization (Δ*V*) /V	Collection coefficient
PP/Li	0.637	2.131
PP/Li-Mg	0.402	1.404
C-PP/Li	0.392	2.140
C-PP/Li-Mg	0.402	2.686
**Asy-PP/Li-Mg**	**0.338**	**4.365**

Table S4. The slope data of the fitting curves (**Figure S19** c, f, i, l and o).

	**PP/Li**	**PP/Li-Mg**	**C-PP/Li**	**C-PP/Li-Mg**	**Asy-PP/Li-Mg**
A	0.08	0.16	0.16	0.20	**0.24**
B	0.07	0.07	0.07	0.13	**0.28**
C	0.06	0.07	0.08	0.09	**0.14**

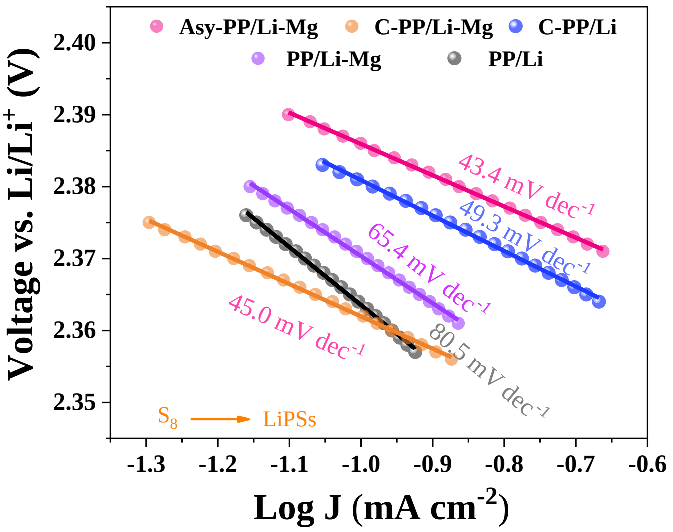

Figure S20 Tafel plots about the conversion reactions of Li_2S_n of different cells.

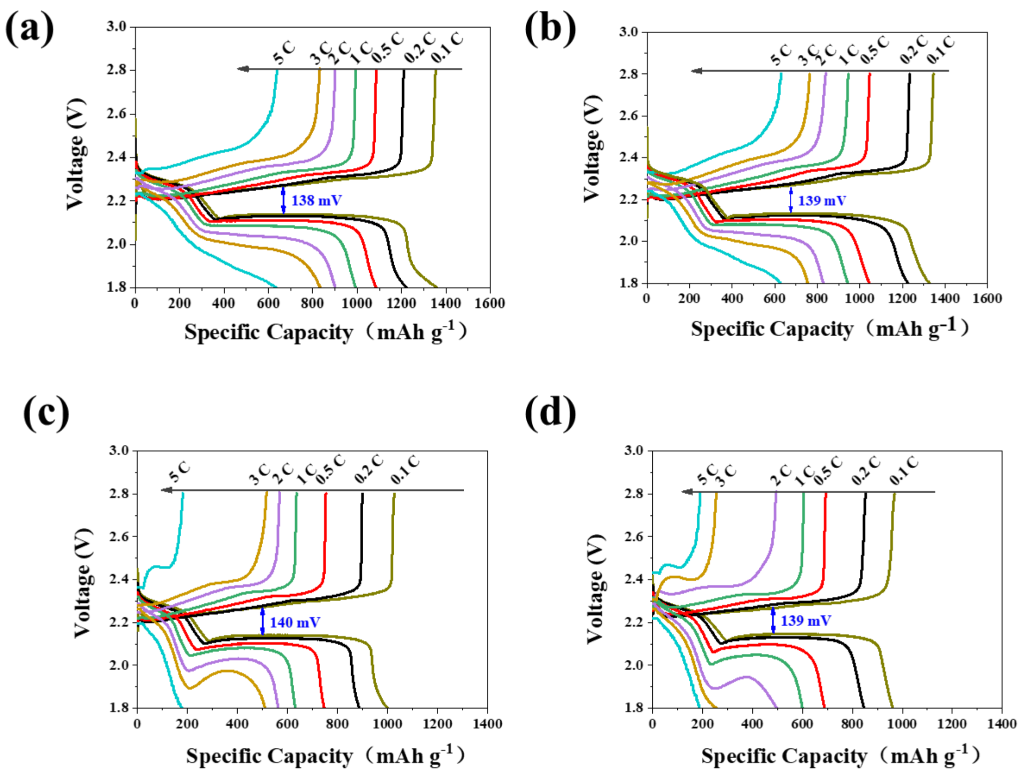

Figure S21 The corresponding galvanostatic discharge-charge curves of rate performance of (a) C-PP/Li-Mg cell (b) C-PP/Li cell, (c)PP/Li-Mg cell and (d)PP/Li.

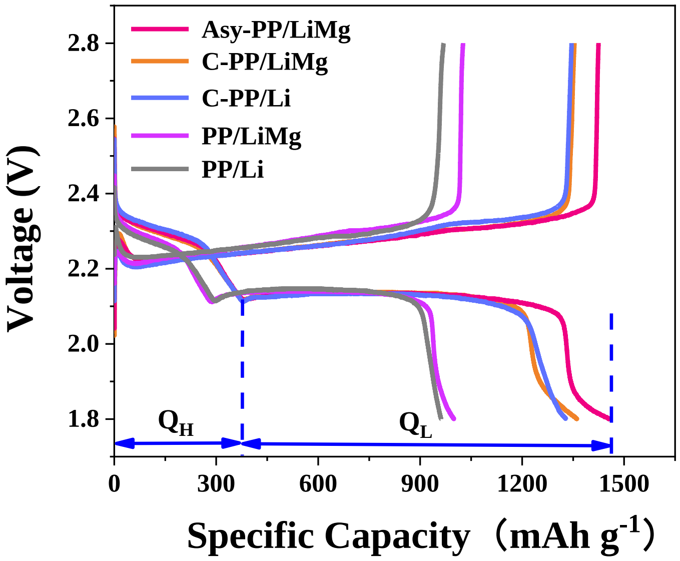

Figure S22 The comparison of the first discharge-charge curves of different cells at 0.1 C.

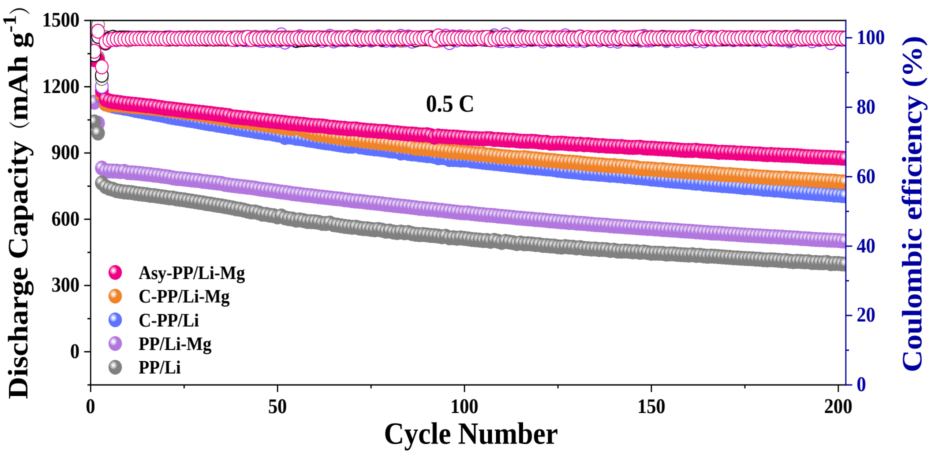

Figure S23 Cycling performance of different cells at 0.5 C (25 °C).

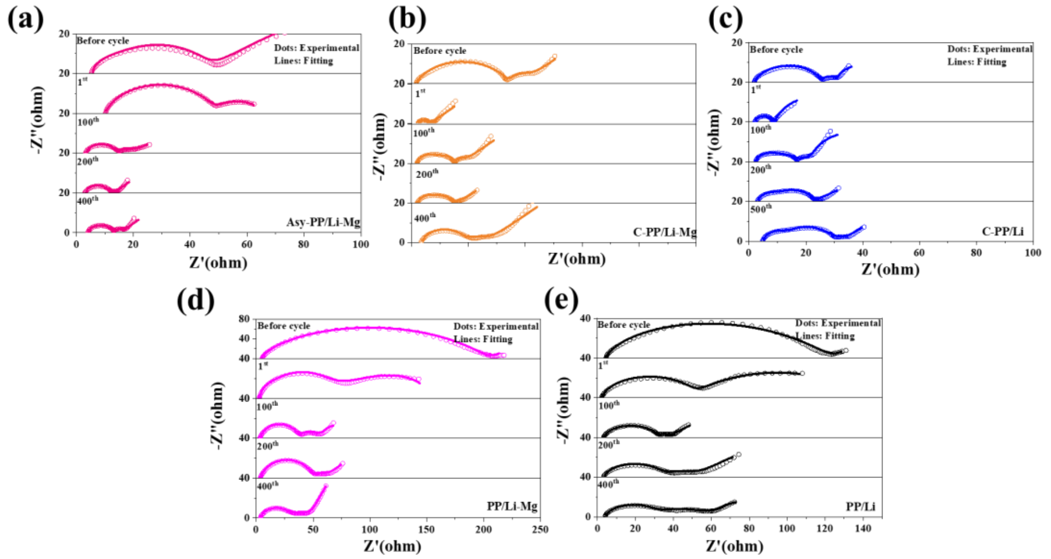

Figure S24. EIS of different Li-S cells before and after different cycles.

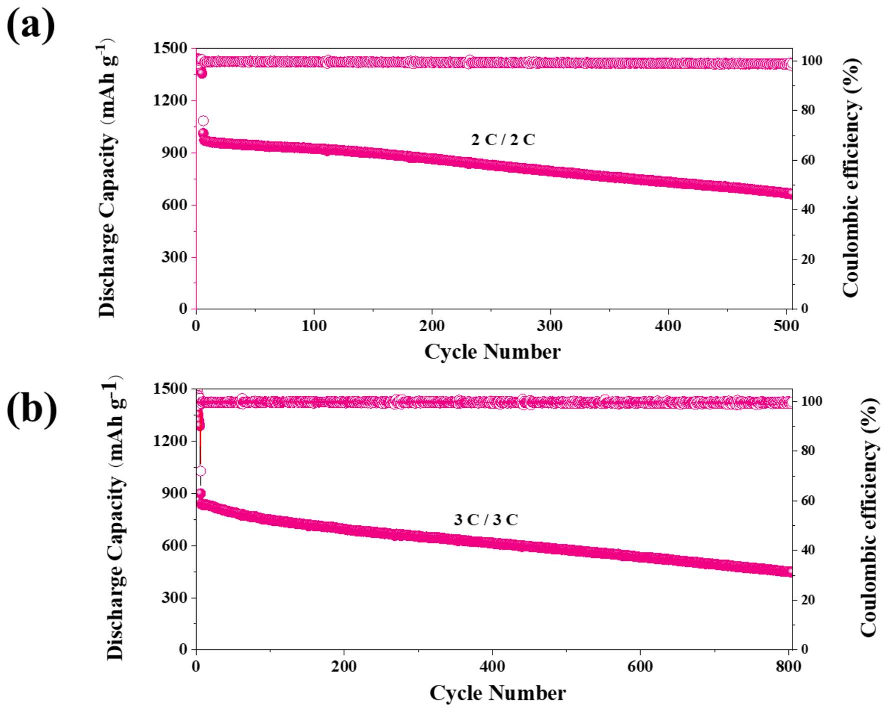

Figure S25 Cycling performance of the Asy-PP/Li-Mg cell at (a) 2 C and (b) 3 C (25 °C).

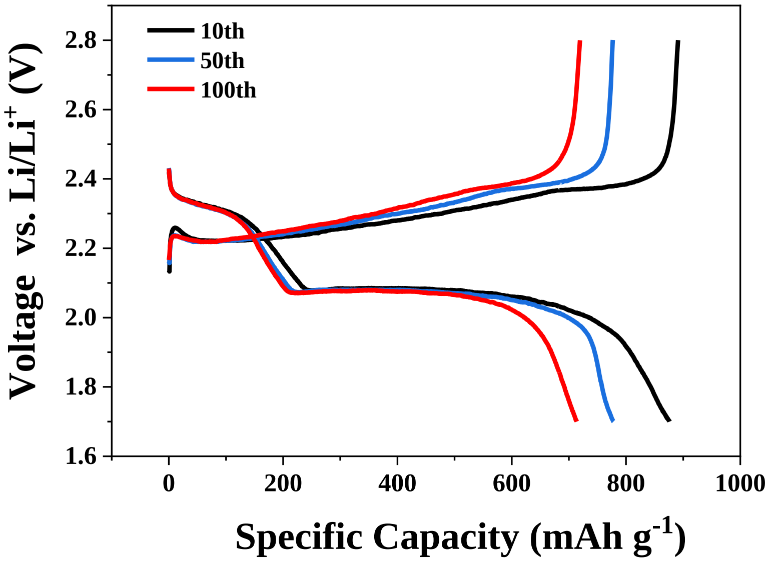

Figure S26. The corresponding galvanostatic discharge-charge curves of the Asy-PP/Li-Mg cell with a high areal sulfur loading of 6.1 mg cm^{-2}.

[1] Kresse, Georg, and D. Joubert. *Phys. Rev. B* **1999**, 59, 1758.

[2] Blöchl, Peter E. *Phys. Rev. B* **1994**, 50, 17953.

[3] Perdew, John P., Kieron Burke, and Matthias Ernzerhof. *Phys. Rev. Lett.* **1996**,77, 3865.

[4] S. Grimme, J. Antony, S. Ehrlich, H. J. T. J. o. c. p. Krieg, *J. Chem. Phys.* **2010**, 132, 154104.
